# Expression of glutamine metabolism-related proteins in thyroid cancer

**DOI:** 10.18632/oncotarget.10682

**Published:** 2016-07-18

**Authors:** Hye Min Kim, Yu Kyung Lee, Ja Seung Koo

**Affiliations:** ^1^ Department of Pathology, Yonsei University College of Medicine, Seoul, South Korea

**Keywords:** thyroid cancer, glutamine, metabolism, stroma, pathology

## Abstract

**Purpose:**

This study aimed to investigate the expression of glutamine metabolism-related protein in tumor and stromal compartments among the histologic subtypes of thyroid cancer.

**Results:**

GLS1 and GDH expression in tumor and stromal compartments were the highest in AC than in other subtypes. Tumoral ASCT2 expression was higher in MC but lower in FC (p < 0.001). In PTC, tumoral GLS1 and tumoral GDH expression was higher in the conventional type than in the follicular variant (p = 0.043 and 0.001, respectively), and in PTC with *BRAF* V600E mutation than in PTC without *BRAF* V600E mutation (p<0.001). Stromal GDH positivity was the independent factor associated with short overall survival (hazard ratio: 21.48, 95% confidence interval: 2.178-211.8, p = 0.009).

**Methods:**

We performed tissue microarrays with 557 thyroid cancer cases (papillary thyroid carcinoma [PTC]: 344, follicular carcinoma [FC]: 112, medullary carcinoma [MC]: 70, poorly differentiated carcinoma [PDC]: 23, and anaplastic carcinoma [AC]: 8) and 152 follicular adenoma (FA) cases. We performed immunohistochemical staining of glutaminolysis-related proteins (glutaminase 1 [GLS1], glutamate dehydrogenase [GDH], and amino acid transporter-2 [ASCT-2]).

**Conclusion:**

Glutamine metabolism-related protein expression differed among the histologic subtypes of thyroid cancer.

## INTRODUCTION

We aimed to investigate the metabolism in thyroid cancer, especially glutamine metabolism in this study. The typical metabolism of malignant tumors can be explained with the Warburg effect. Most cancer cells produce energy not via a low rate of glycolysis based on oxidation of pyruvate in mitochondria but via a high rate of glycolysis based on lactic acid fermentation in cytosol [1]. Although aerobic glycolysis is one of the most important metabolic pathways, the flexibility of the metabolic system is a major obstacle in cancer treatment. Glutamine metabolism is a critical pathway in cancer cell metabolism [2]. In previous studies, cancer cells have been shown to metabolize glutamine more than other amino acids [3, 4]. Therefore, glutamine metabolism is an important metabolic phenotype of proliferating cancer cells, because it satisfies two important elements of the proliferating cancer cells, i.e., ATP production and supply of intermediates for macromolecular synthesis [2]. Furthermore, in glutamine metabolic pathway, the interaction between cancer cells and stromal cells is present. It was reported that ammonia, which is produced by tumor cell glutaminolysis migrates into the stroma, leading to increased autophagy of stromal cells. Consequently, glutamine was reported to be produced as the product of autophagy activity, travel back to tumor cells and utilized in glutamine metabolism [5-9].

The three proteins that play an important role in glutamine metabolic pathway are the amino acid transporter-2 (ASCT2), the transporter involved in the movement of glutamine into the cell that would be consumed by cancer cells [10]; glutaminase 1 (GLS1), the enzyme that converts glutamine to glutamate [11]; and glutamate dehydrogenase (GDH), the enzyme that converts glutamate to α-ketoglutarate to be used in the tricarboxylic acid cycle [12].

Thyroid cancer is common, accounting for approximately 1.5% of the population [13]. The representative subtypes are papillary thyroid carcinoma (PTC), follicular carcinoma (FC), medullary carcinoma (MC), poorly differentiated carcinoma (PDC), and anaplastic carcinoma (AC). The cell origin, clinical manifestation, metastatic pattern, and clinical prognosis are different depending on the histologic subtype [14]. In previous studies of various tumors, differences in the expression of metabolism-related proteins were reported among the histological subtypes [15-18]. Thus, such differences can be expected in thyroid cancer as well; however, much research on this topic is required. Therefore, the purpose of this study was to investigate the expression of glutamine metabolism-related protein in tumor and stromal compartments depending on the subtype of thyroid cancer and its implications on patient prognosis.

## RESULTS

### Basal characteristics of patients with thyroid cancer

This study included 557 cases of thyroid cancer, as follows: 344 cases of PTC, 112 cases of FC, 70 cases of MC, 23 cases of PDC, and 8 cases of AC. The basal characteristics of the patients with PTC are shown in Supplementary Table 2. According to the histologic subtype of PTC, the samples were classified as the conventional type PTC (304 cases) and the follicular variant PTC (40 cases). A higher proportion of follicular variant PTC had expanding tumor margin (p = 0.002). Among the PTC cases, 238 cases (69.18%) had *BRAF* V600E mutation. A higher proportion of these cases had infiltrative tumor margin (p = 0.004) but the follicular variant occupied a lower proportion (p < 0.001) (Supplementary Table 2). FC included 99 cases of the minimally invasive type and 13 cases of the widely invasive type. The widely invasive type had a higher proportion of large tumor size (p = 0.040), vascular invasion (p = 0.028), extrathyroidal involvement (p < 0.001), and distant metastasis (p = 0.003) (Supplementary Table 3). The basal characteristics of patients with MC, PDC, and AC are shown in Supplementary Table 4.

### Expression of glutamine metabolism-related proteins in thyroid cancer

**Figure 1:**
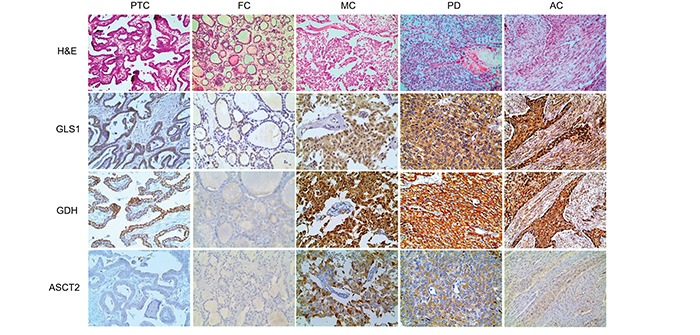
Expression of glutamine metabolism-related proteins according to the histologic subtype of thyroid cancer The expression of tumoral GLS1 and tumoral GDH is higher in anaplastic carcinoma, but absent in follicular carcinoma; stromal GLS1 and stromal GDH expression are higher in anaplastic carcinoma. Tumoral ASCT2 expression is higher in medullary carcinoma, but absent in follicular carcinoma. The photos of GLS1, GDH, and ASCT2 expression are obtained from the same case of each subtype of thyroid cancer. GLS1; glutaminase 1, GDH; glutamate dehydrogenase, ASCT2; amino acid transporter-2.

We investigated the expression of glutamine metabolism-related proteins in thyroid cancer. GLS1 and GDH were expressed in both tumor cells and stroma, but ASCT2 was expressed only in the tumor cells. The expression of glutamine metabolism-related proteins was different according to the histologic subtype of thyroid cancer (Figure 1 and Table 1), where the expression of tumoral GLS1 and tumoral GDH was higher in AC but lower in FC. Stromal GLS1 expression was observed only in AC, and stromal GDH expression was higher in AC. Tumoral ASCT2 expression was higher in MC but lower in FC (p < 0.001).

**Table 1:** Expression of glutamine metabolism-related proteins according to the histologic subtype of thyroid cancer

Parameters	Total N= 557(%)	PTC n= 344(%)	FC n= 112(%)	MC n= 70(%)	PDC n= 23(%)	AC n= 8(%)	p-value
GLS1 (T)							<0.001
Negative	247 (44.3)	141 (41.0)	70 (62.5)	27 (38.6)	9 (39.1)	0 (0.0)	
Positive	310 (55.7)	203 (59.0)	42 (37.5)	43 (61.4)	14 (60.9)	8 (100.0)	
GLS1 (S)							<0.001
Negative	554 (99.5)	344 (100.0)	112 (100.0)	70 (100.0)	23 (100.0)	5 (62.5)	
Positive	3 (0.5)	0 (0.0)	0 (0.0)	0 (0.0)	0 (0.0)	3 (37.5)	
GDH (T)							<0.001
Negative	105 (18.9)	43 (12.5)	48 (42.9)	11 (15.7)	3 (13.0)	0 (0.0)	
Positive	452 (81.1)	301 (87.5)	64 (57.1)	59 (84.3)	20 (87.0)	8 (100.0)	
GDH (S)							<0.001
Negative	549 (98.6)	341 (99.1)	112 (100.0)	70 (100.0)	22 (95.7)	4 (50.0)	
Positive	8 (1.4)	3 (0.9)	0 (0.0)	0 (0.0)	1 (4.3)	4 (50.0)	
ASCT2 (T)							<0.001
Negative	459 (82.4)	289 (84.0)	103 (92.0)	43 (61.4)	17 (73.9)	7 (87.5)	
Positive	98 (17.6)	55 (16.0)	9 (8.0)	27 (38.6)	6 (26.1)	1 (12.5)	

PTC; papillary thyroid carcinoma, FC; follicular carcinoma, MC; medullary carcinoma, PDC; poorly differentiated carcinoma, AC; anaplastic carcinoma, GLS1; glutaminase 1, GDH; glutamate dehydrogenase, ASCT2; amino acid transporter-2

The results of the correlation analysis of the expression of GLS1, GDH, and ASCT showed significant quantitative correlation between GDH and GLS1 (r = 0.364), between GDH and ASCT2 (r = 0.174), between GLS1 and ASCT2 (r = 0.213), and between GDH(S) and GLS1(S) (r = 0.403; all p < 0.001; Table 2). Furthermore, we performed a correlation analysis between the subtype of thyroid cancer with the expression of GLS1, GDH, and ASCT. As a result, in PTC, statistically significant correlation was shown between GLS1 and GDH (r=0.328, p<0.001), between GLS1 and ASCT2 (r=0.251, p<0.001), and between GDH and ASCT2 (r=0.165, p=0.002). The same result was also shown in FC, with a significant correlation between GLS1 and GDH (r=0.373, p<0.001), between GLS1 and ASCT2 (r=0.246, p=0.010), and between GDH and ASCT2 (r=0.190, p=0.046). In addition, the correlation between GLS1 and GDH (r=0.030, p=0.012) was shown in MC and between GLS1 and ASCT2 (r=0.476, p=0.025) in PDC were noted.

**Table 2:** Correlation among the expressions of glutamine metabolism-related proteins

Parameters	GLS1	GDH	ASCT2	GLS1 (S)
GLS1				
Correlation coefficient				
p-value				
GDH				
Correlation coefficient	0.364			
p-value	**<0.001**			
ASCT2				
Correlation coefficient	0.213	0.174		
p-value	**<0.001**	**<0.001**		
GLS1 (S)				
Correlation coefficient	0.066	0.035	-0.034	
p-value	0.121	0.403	0.423	
GDH (S)				
Correlation coefficient	0.077	0.058	0.023	0.403
p-value	0.068	0.170	0.580	**<0.001**

### Expression of glutamine metabolism-related proteins according to the histologic subtype of thyroid cancer

**Figure 2:**
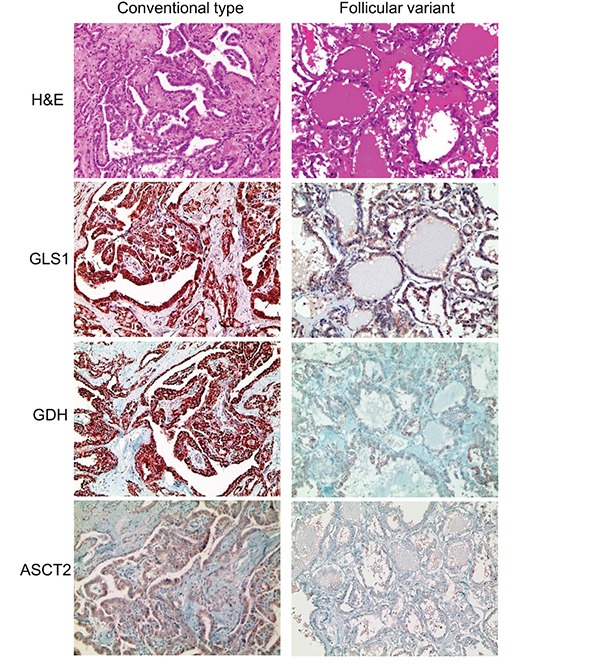
Expression of glutamine metabolism-related proteins according to the histologic subtype of papillary thyroid cancer (PTC) Tumoral GLS1 and tumoral GDH expression are higher in the conventional type of PTC than in the follicular variant of PTC. GLS1; glutaminase 1, GDH; glutamate dehydrogenase.

**Figure 3:**
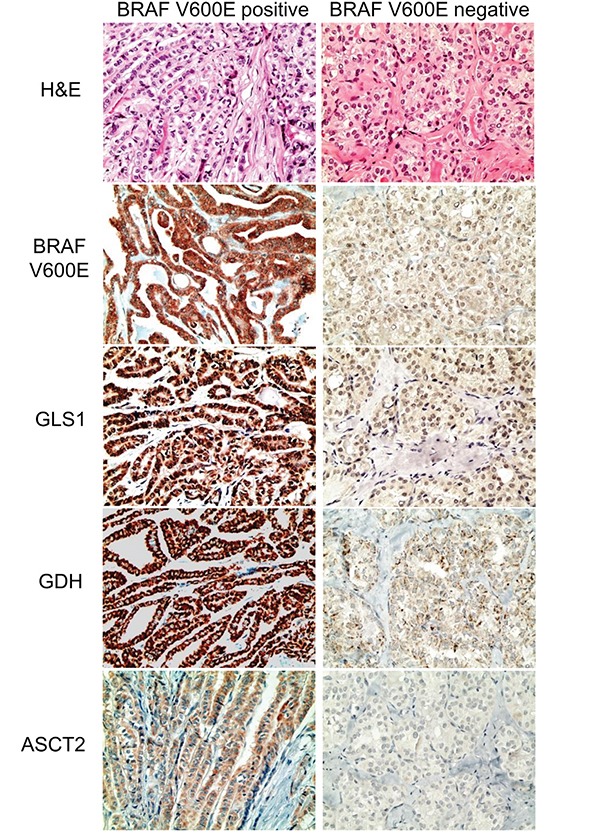
Expression of glutamine metabolism-related proteins according to the BRAF V600E mutation status in papillary thyroid cancer Papillary thyroid cancer with the BRAF V600E mutation shows higher expression of tumoral GLS1, tumoral GDH, and tumoral ASCT2. GLS1; glutaminase 1, GDH; glutamate dehydrogenase, ASCT2; amino acid transporter-2

**Figure 4:**
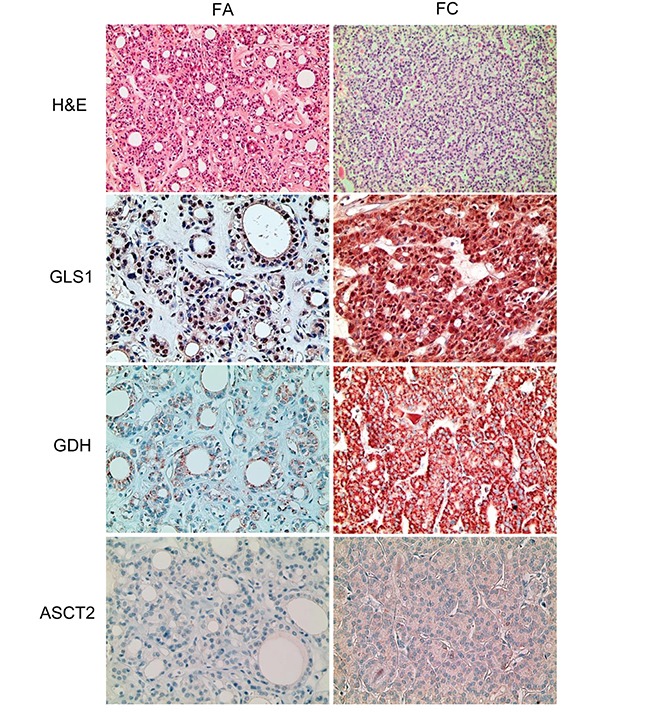
Expression of glutamine metabolism-related proteins in follicular neoplasms Tumoral GLS1 and tumoral GDH expression are higher in follicular carcinoma than in follicular adenoma. GLS1; glutaminase 1, GDH; glutamate dehydrogenase.

We investigated the expression of glutamine metabolism-related proteins according to the histologic subtype of thyroid cancer. First, in PTC, tumoral GLS1 and tumoral GDH expression was higher in the conventional type than in the follicular variant (p = 0.024 and p < 0.001, respectively) (Figure 2 and Table 3). In addition, PTC with the *BRAF* V600E mutation showed higher expression of tumoral GLS1, tumoral GDH, and tumoral ASCT2 (p < 0.001) (Figure 3 and Table 3). Secondly, in follicular neoplasms, tumoral GLS1 and tumoral GDH expression was higher in FC than in FA (p = 0.021 and 0.001, respectively) (Figure 4 and Table 4). Thirdly, in FC, the expression of glutamine metabolism-related proteins showed no significant difference between the minimally invasive type and the widely invasive type (Table 5).

**Table 3:** Expression of glutamine metabolism-related proteins according to the histologic subtype and BRAF V600E mutation status of papillary thyroid carcinoma

Parameters	Total N=344 (%)	Histologic subtype		p-value	BRAF V600E mutation status		p-value
		Conventional type n= 304 (%)	Follicular variant n= 40 (%)		No mutation n= 106 (%)	Mutation n= 238 (%)	
GLS1 (T)				**0.024**			**<0.001**
Negative	141 (41.0)	118 (38.8)	23 (57.5)		58 (54.7)	83 (34.9)	
Positive	203 (59.0)	186 (61.2)	17 (42.5)		48 (45.3)	155 (65.1)	
GDH (T)				**<0.001**			**<0.001**
Negative	43 (12.5)	28 (9.2)	15 (37.5)		26 (24.5)	17 (7.1)	
Positive	301 (87.5)	276 (90.8)	25 (62.5)		80 (75.5)	221 (92.9)	
GDH (S)				1.000			0.246
Negative	341 (99.1)	301 (99.0)	40 (100.0)		106 (100.0)	235 (98.7)	
Positive	3 (0.9)	3 (1.0)	0 (0.0)		0 (0.0)	3 (1.3)	
ASCT2 (T)				**0.041**			**<0.001**
Negative	289 (84.0)	251 (82.6)	38 (95.0)		102 (96.2)	187 (78.6)	
Positive	55 (16.0)	53 (17.4)	2 (5.0)		4 (3.8)	51 (21.4)	

GLS1; glutaminase 1, GDH; glutamate dehydrogenase, ASCT2; amino acid transporter-2

**Table 4:** Expression of glutamine metabolism-related proteins in follicular neoplasms

Parameters	Total N=264 (%)	FA n=152 (%)	FC n=112 (%)	p-value
GLS1 (T)				0.021
Negative	185 (70.1)	115 (75.7)	70 (62.5)	
Positive	79 (29.9)	37 (24.3)	42 (37.5)	
GDH (T)				0.001
Negative	144 (54.5)	96 (63.2)	48 (42.9)	
Positive	120 (45.5)	56 (36.8)	64 (57.1)	
ASCT2 (T)				0.156
Negative	249 (94.3)	146 (96.1)	103 (92.0)	
Positive	15 (5.7)	6 (3.9)	9 (8.0)	

FA; follicular adenoma, FC: follicular carcinoma, GLS1; glutaminase 1, GDH; glutamate dehydrogenase, ASCT2; amino acid transporter-2

**Table 5:** Expression of glutamine metabolism-related proteins according to the histologic subtype of follicular carcinoma (FC)

Parameters	Total N=112 (%)	FC, minimally invasive type n=99 (%)	FC, widely invasive type n=13 (%)	p-value
GLS1 (T)				0.493
Negative	70 (62.5)	63 (63.6)	7 (53.8)	
Positive	42 (37.5)	36 (36.4)	6 (46.2)	
GDH (T)				0.394
Negative	48 (42.9)	41 (41.4)	7 (53.8)	
Positive	64 (57.1)	58 (58.6)	6 (46.2)	
ASCT2 (T)				0.257
Negative	103 (92.0)	90 (90.9)	13 (100.0)	
Positive	9 (8.0)	9 (9.1)	0 (0.0)	

FC: follicular carcinoma, GLS1; glutaminase 1, GDH; glutamate dehydrogenase, ASCT2; amino acid transporter-2

### Correlation between the expression of glutamine metabolism-related proteins and clinicopathologic factors

**Figure 5:**
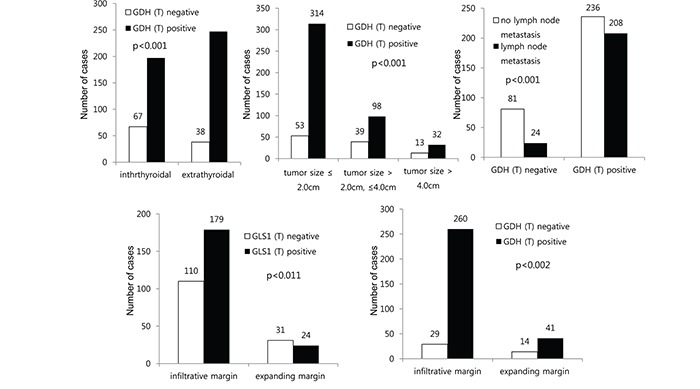
Correlation between the expressions of glutamine metabolism-related proteins and clinicopathologic factors in thyroid cancer and papillary thyroid carcinoma

We investigated the correlation between the expression of glutamine metabolism-related proteins and the clinicopathologic factors in patients with thyroid cancer except for AC. Tumoral GDH positivity was associated with extrathyroidal extension, small tumor size, and lymph node metastasis (p < 0.001 for all). In PTC, the expression of tumoral GLS1 and tumoral GDH was associated with an infiltrative tumor margin (p = 0.011 and 0.002, respectively) (Figure 5).

### Influence of the expression of glutamine metabolism-related proteins on patient prognosis

We investigated the effect of the expression of glutamine metabolism-related proteins on prognosis through univariate analysis and multivariate Cox analysis. In univariate analysis, shorter disease-free survival was associated with stromal GDH expression (p < 0.001) (Table 6). In multivariate Cox analysis, shorter disease-free survival was associated with lymph node metastasis (hazard ratio: 6.021, 95% confidence interval [CI]: 1.352-26.82, p = 0.019), and shorter overall survival was associated with old age (≥45 years, hazard ratio: 25.26, 95% CI: 3.047-209.4, p = 0.003), lymph node metastasis (hazard ratio: 4.466, 95% CI: 1.352-14.76, p = 0.014) and stromal GDH positivity (hazard ratio: 21.48, 95% CI: 2.178-211.8, p = 0.009) (Table 7).

**Table 6:** Univariate analysis of the influence of glutamine-related protein expression in thyroid cancer on disease-free and overall survival by the log-rank test

Parameter	Number of patients*/recurrence/death	Disease-free survival		Overall survival	
		Mean survival (95% CI) months	*P*-value	Mean survival (95% CI) months	*P*-value
GLS1 (T)			0.141		0.938
Negative	247/22/13	101 (98-105)		106 (103-108)	
Positive	302/17/17	106 (103-108)		106 (103-108)	
GDH (T)			0.470		0.896
Negative	105/9/5	101 (96-107)		106 (103-110)	
Positive	444/30/25	104 (102-107)		106 (104-108)	
GDH (S)			0.110		**<0.001**
Negative	545/38/28	104 (102-106)		107 (105-108)	
Positive	4/1/2	68 (24-112)		54 (13-95)	
ASCT2 (T)			0.677		0.217
Negative	452/33/22	104 (101-106)		107 (105-109)	
Positive	97/6/8	103 (98-108)		102 (96-107)	

* cases of AC were excluded. GLS1; glutaminase 1, GDH; glutamate dehydrogenase, ASCT2; amino acid transporter-2

**Table 7:** Multivariate analysis of factors influencing survival of patients with thyroid cancer *

Included parameters	Disease-free survival			Overall survival		
	Hazard ratio	95% CI	*P*-value	Hazard ratio	95% CI	*P*-value
Age (years)			0.631			**0.003**
<45 versus ≥45	1.263	0.488-3.272		25.26	3.047-209.4	
Sex			0.668			0.402
Male versus Female	1.265	0.433-3.690		1.564	0.550-4.449	
Tumor size (cm)			0.798			0.402
≤2.0 versus >2.0	1.147	0.400-3.288		1.564	0.550-4.449	
Tumor extension			0.352			0.504
Intrathyroidal versus Extrathyroidal	0.631	0.239-1.663		0.688	0.229-2.061	
LN metastasis			**0.019**			**0.014**
No versus Yes	6.021	1.352-26.82		4.466	1.352-14.76	
GLS1 (T)			0.273			0.422
Negative versus positive	0.587	0.226-1.522		0.676	0.261-1.757	
GDH (S)			n/a			**0.009**
Negative versus positive	n/a	n/a		21.48	2.178-211.8	

* cases of AC were excluded. GLS1; glutaminase 1, GDH; glutamate dehydrogenase

## DISCUSSION

In this study, GLS1 and GDH showed higher expression in both tumor cells and stroma in AC, but it was lower in FC. In general, the higher tumor aggressiveness, the higher metabolic activity and glutamine metabolism-related protein - GLS1, GDH, and ASCT2 - has been reported to be associated with tumor aggressive factor in other tumors [19-21]. There are several possible reasons for the higher expression of glutamine metabolism-related proteins in AC than in other subtypes: different tumor cell proliferative activity, high metastatic potential, and the impact of oncogenic driver. AC should show the highest metabolic activity, as metabolic activity was reported to be more than three times greater in AC than in other subtypes of thyroid cancer [22]. AC also has high metastatic potential, with distant metastasis reported at diagnosis in 46% of cases [23], and in tumor metastasis, glutamine metabolism has been reported to play an important role [24]. Finally, unlike other subtypes of thyroid cancer, AC has been shown to have increased HER-2 expression [25] and activation of Wnt β-catenin pathway [26]. HER-2 and β-catenin pathway have been reported to be associated with increased glutamine metabolism [27, 28].

One interesting point is that in PDC, a highly aggressive type of tumor similar to AC, the expression of GLS1 and GDH was not significantly higher than that of other subtypes, unlike AC. The different expressions in PDC and AC can be explained by the genetic change between the two subtypes. In previous studies, one major difference was that TP53 mutation, which is known to be associated with metabolism, was shown in 26% of PDC samples as opposed to 60% of AC samples [29-31]. Glut-1 and Glut-4, key proteins in glycolysis, were reported to be inhibited by wild type p53 [32]. Considering that TP53 mutation neutralizes this interaction, AC was reported to exhibit TP53 and Glut-1 overexpression [33]. An association between TP53 mutation and metabolism was also shown, potentially explaining the different GLS1 and GDH expressions in PDC and AC in this study. Furthermore, p53 immunohistochemical staining was performed in PDC and AC to evaluate the association with the expressions of glutamine metabolism-related proteins. As a result, p53 positivity showed an association with stromal GDH expression (p=0.029), tumoral GLS1 (p=0.054), and stromal GLS1 (p=0.055), further supporting the relationship between p53 positivity and the expressions of glutamine metabolism-related proteins (Supplementary Table 5). Further research using next-generation sequencing analyzed the genetic alterations between PDC and AC. As a result, several genetic changes were coincided in PDC and AC. However, each type has been reported as having its own specific genetic background [34].

In particular, the expression in AC was high in stroma as well as in tumor cells. In breast cancer, glutamine metabolism-related protein expression in stroma was similar to such expression in tumor cells [21]. Metabolic interaction could exist between tumoral and stromal cells, such as cancer-associated fibroblasts, according to the cancer types [7, 35-38]. In addition, the interaction has been reported to exist in glutamine metabolic pathway. Previous studies have shown a vicious cycle wherein ammonia (a byproduct of tumor cell glutaminolysis) diffuses into the stroma, induces autophagy, produces glutamine as a product of autophagy activity, and is passed back to tumor cells [5-9]. In one study, when the MCF-7 cell line was co-cultured with fibroblasts, the expression of GLS, GDH, and SLC6A14 (glutamine importer) in tumor cells increased and glutamine neosynthesis decreased compared to when the cell line was cultured alone. The transmission of glutamine from stromal cells to tumor cells has demonstrated, and in the case, the cell proliferation has reported to be increased [9]. Therefore, the interaction between tumor cells and stromal cells in the glutamine metabolic pathway promotes tumor cell proliferation and growth. The increased glutamine metabolic activity in tumor cells and stromal cells in AC contributes to tumor aggressiveness. In addition, tumoral GLS1 and GDH expression was the highest in conventional type PTC followed by follicular variant PTC, FC, and FA; therefore, GLS1 and GDH were proven to show lower expression with more follicular differentiation. As the expression of metabolic pathway-related protein such as Glut-1 has been reported to be different according to tumor differentiation in various human cancers [39, 40], such a phenomenon in thyroid cancer can seem possible.

PTC with the *BRAF* V600E mutation showed a higher expression of glutamine metabolism-related proteins. The BRAF V600E mutation is associated with extra-thyroidal extension, advanced TNM stage, lymph node metastasis, multifocality, and recurrence in a meta-analysis study [41]. Because PTC with the BRAF V600E mutation has aggressive tumor biology, it can be suggested that it shows higher expression of glutamine metabolism-related proteins. In addition, PTC with the BRAF V600E mutation has been reported to show increased glucose metabolism [42]. One possible mechanism is that the BRAF mutation is associated with the activation of mitogen-activated protein kinase downstream molecules such as c-MYC and HIF-1a; therefore, glucose metabolism increases. Furthermore, cell proliferation in melanoma cells with BRAF mutations has been reported to rely on glutamine metabolism [43]. Accordingly, the association between PTC with the *BRAF* V600E mutation and increased glutamine metabolism is supported.

The expression of ASCT2 was higher in MC. The association with the *MYC* gene can be considered a possible mechanism. In previous studies, MYC expression has been reported in MC [44-46]. The *MYC* gene has been reported to increase the expression of ASCT2 by binding to the promoter element of the glutamine transporter [47]; hence, the higher ASCT2 expression in MC can be explained.

Stromal GDH expression was an independent prognostic factor in thyroid cancer. In previous studies, GDH expression was shown to be associated with poor prognosis in colon cancer [48], carcinoma of unknown primary [49], and breast phyllodes tumor [20]. The compartment with GDH expression differed depending on the tumor. Stromal GDH expression in breast phyllodes tumors [20] and tumoral GDH expression in carcinoma of unknown primary [49] have been reported to be associated with patient prognosis. An analysis of colon cancer did not divide the compartment between tumor cells and stroma; therefore, GDH expression could not be evaluated in this case [48]. Likewise, the influence of GDH on prognosis differs according to the expressed compartments.

In this study, we showed positive correlation between the expression of GLS1, GDH and ASCT2. This positive correlation was observed when targeting not only all subtypes of thyroid cancer but also PTC and FC and a partial correlation was also shown in MC and PDC. It suggests the role of GLS1, GDH, and ASCT2 evaluation by immunohistochemical staining could possibly reflect the glutamine metabolism activity.

The results suggest that the glutamine metabolism pathway can be used as a possible target for therapy in thyroid cancer. The potential candidates for target therapy among thyroid cancer are radioiodine refractory differentiated thyroid cancer, PDC, AC, and MC. Targeted therapies that is currently used in thyroid cancer are tyrosine kinase inhibitors (TKIs) and monoclonal antibodies (mAbs), which inhibits the tyrosine kinase activity that is essential in the pathogenesis of thyroid cancer [50]. However, clinical trials regarding TKIs and mAbs had shown unsatisfactory results, only with a partial response rate ranging from 2 to 45% [51-54]. This might be attributable by the drug resistance, cytostatic action of TKIs and drug toxicity [50], necessitating novel target agent to overcome these limitations. The possible strategy is to reduce glutamine metabolic enzyme activity or reduce glutamine uptake. GLS1 inhibitors including BPTES [55-57], 968 [58-60], and CB-839 [61, 62] are under preclinical and clinical trials for the treatment of various tumors. BenSer as an ASCT2 inhibitor has been reported to inhibit the proliferation of melanoma cells [63]. In particular, radioiodine refractory thyroid tumor might be suitable for targeted therapy since it shows positive scan in 18FDG-PET [64]. Therefore, the inhibition of metabolic pathway might be used as an alternative effective therapeutic option, which requires further studies.

In conclusion, the expression of glutamine metabolism-related proteins in thyroid cancer was found to differ according to histologic subtype. GLS1 and GDH expression was high in both tumor cells and stroma of AC. ASCT2 expression was high in MC. In addition, tumoral GLS1 and tumoral GDH expression was high in PTC with the *BRAF* V600E mutation. Therefore, the glutamine metabolism pathway may be a possible target for therapy in thyroid cancer.

## MATERIALS AND METHODS

### Patient selection

For PTC, we included patients who underwent surgery at Severance Hospital between January 2012 and December 2013. For other subtypes, we included patients who underwent surgery at Severance Hospital between January 2000 and December 2014. Patients treated with neoadjuvant chemotherapy were excluded. This study was approved by the Institutional Review Board (IRB) of Yonsei University Severance Hospital. Consent forms of patients were exempt by IRB. All cases were retrospectively reviewed by a thyroid pathologist (Koo JS), and histologic review was performed by using hematoxylin and eosin-stained slides. Clinicopathologic data were obtained from the patients' medical records; they included age at diagnosis, disease recurrence, metastasis, current status, and length of follow-up. The tumor size, tumor margin (infiltrative or expanding), extent (confined to the thyroid parenchyma or with extrathyroidal spread), and the status of metastatic lymph nodes were also noted from review of the slides and the surgical pathology reports.

### Tissue microarray analysis

Representative areas were selected on hematoxylin-eosin-stained slides, and the corresponding spot was marked on the surface of the matching paraffin block. Core biopsies (3 mm) were taken from selected areas and placed into a 6 × 5 recipient block. Two tissue cores were extracted from each case to minimize extraction bias. Each tissue core was assigned a unique tissue microarray location number that was linked to a database containing other clinicopathologic data.

### Immunohistochemistry

Antibodies used for immunohistochemistry are listed in Supplementary Table 1. All immunohistochemistry was performed on formalin-fixed, paraffin-embedded tissue sections by using an automatic immunohistochemistry staining device (Benchmark XT, Ventana Medical System, Tucson, AZ, USA). Briefly, 5-μm-thick formaldehyde-fixed paraffin-embedded tissue sections were transferred onto adhesive slides and dried at 62°C for 30 minutes. Standard heat epitope retrieval was performed for 30 minutes in ethylene diamine tetraacetic acid, pH 8.0, in the autostainer. The samples were then incubated with primary antibodies. After incubation with primary antibodies, the sections were subsequently incubated with biotinylated anti-mouse immunoglobulins, peroxidase-labeled streptavidin (LSAB kit, DakoCytomation), and 3,3'-diaminobenzidine. Negative control samples were processed without the primary antibody. Positive control tissue was used as per the manufacturer's recommendation. All slides were counterstained with Harris hematoxylin.

### Interpretation of immunohistochemical staining

Immunohistochemical markers were assessed under light microscopy with x200 magnification in the entire core of the tissue microarray. The expression of glutamine metabolism-related proteins was evaluated semi-quantitatively according to a previously reported method [65]. Tumoral and stromal staining was assessed as 0: negative or weak immunostaining in <1% of the tumor/stroma, 1: focal expression in 1-10% of the tumor/stroma, 2: positive in 11%-50% of the tumor/stroma, and 3: positive in 51%-100% of the tumor/stroma. The score of ≥2 was defined as positive. BRAF V600E staining was considered positive when more than 20% tumor cells were positive [66]. Immunohistochemical staining was interpreted independently by two pathologists (KHM and KJS). In cases of discrepancy between the pathologists, the final result was determined through discussion under the multi-view microscopy.

### Statistical analysis

Data were analyzed using SPSS for Windows, Version 12.0 (SPSS Inc., Chicago, IL, USA). For determination of statistical significance, the Student *t*-test and Fisher exact tests were used for continuous and categorical variables, respectively. The correlation between the expression of GLS1, GDH, and ASCT was analyzed using Kendall's method. In the case of analyzing data with multiple comparisons, a corrected p-value with the application of the Bonferroni multiple comparison procedure was used. Statistical significance was set at p < 0.05. Kaplan-Meier survival curves and log-rank statistics were employed to evaluate time to tumor recurrence and overall survival. Multivariate regression analysis was performed using the Cox proportional hazards model.

## SUPPLEMENTARY FIGURES AND TABLES




