# Supplementary material for: Expression of glutamine metabolism-related proteins in thyroid cancer
Source: Oncotarget. 2016 Jul 18;7(33):53628–41. doi: 10.18632/oncotarget.10682 (PMC5288210; doi:10.18632/oncotarget.10682)
Supplement: Supplementary file 1 [file oncotarget-07-53628-s001.pdf]

Expression of glutamine metabolism-related proteins in thyroid cancer

SUPPLEMENTARY TABLES

Supplementary Table S1: Source, clone, and dilution of antibodies used in this study

Antibody	Clone	Dilution	Company
GLS1	Polyclonal	1:50	Abcam, Cambridge, UK
GDH	Polyclonal	1:100	Abcam, Cambridge, UK
ASCT2	Polyclonal	1:100	Abcam, Cambridge, UK
BRAF V600E	VE1	1:50	Ventana, Tucson, AZ, USA

GLS1; glutaminase 1, GDH; glutamate dehydrogenase, ASCT2; amino acid transporter-2

Supplementary Table S2: Basal characteristics of patients with papillary thyroid carcinoma

See Supplementary File 1

Supplementary Table S3: Basal characteristics of patients with follicular carcinoma (FC)

Parameters	Total N=112 (%)	FC, minimally invasive type n=99 (%)	FC, widely invasive type n=13 (%)	p-value
Age (years)				0.255
<45	51 (45.5)	47 (47.5)	4 (30.8)	
≥45	61 (54.5)	52 (52.5)	9 (69.2)	
Sex				0.233
Male	28 (25.0)	23 (23.2)	5 (38.5)	
Female	84 (75.0)	76 (76.8)	8 (61.5)	
Tumor size (cm)				0.040
≤2.0	34 (30.4)	34 (34.3)	0 (0.0)	
>2.0, ≤4.0	49 (43.8)	41 (41.4)	8 (61.5)	
>4.0	29 (25.9)	24 (24.2)	5 (38.5)	
Capsular invasion				0.147
No	14 (12.5)	14 (14.1)	0 (0.0)	
Yes	98 (87.5)	85 (85.9)	13 (100.0)	
Vascular invasion				0.028
No	66 (58.9)	62 (62.6)	4 (30.8)	
Yes	46 (41.1)	37 (37.4)	9 (69.2)	
Tumor extension				<0.001
Intrathyroidal	95 (84.8)	89 (89.9)	6 (46.2)	
Extrathyroidal	17 (15.2)	10 (10.1)	7 (53.8)	
LN metastasis				0.220
No	110 (98.2)	98 (99.0)	12 (92.3)	
Yes	2 (1.8)	1 (1.0)	1 (7.7)	
Distant metastasis				0.003
No	101 (90.2)	93 (93.9)	8 (61.5)	
Yes	11 (9.8)	6 (6.1)	5 (38.5)	

Supplementary Table S4: Basal characteristics of patients with medullary carcinoma (MC), pooly differentiated carcinoma (PDC), and anaplastic carcinoma (AC)

Parameters	MC, n= 70 (%)	PDC, n= 23 (%)	AC, n= 8 (%)
Age (years)			
<45	21 (30.0)	4 (17.4)	0 (0.0)
≥45	49 (70.0)	19 (82.6)	8 (100.0)
Sex			
Male	22 (31.4)	10 (43.5)	1 (12.5)
Female	48 (68.6)	13 (56.5)	7 (87.5)
Tumor size (cm)			
≤2.0	53 (75.7)	8 (34.8)	0 (0.0)
>2.0, ≤4.0	14 (20.0)	9 (39.1)	1 (12.5)
>4.0	3 (4.3)	6 (26.1)	7 (87.5)
Tumor margin			
Infiltrative	45 (64.3)	17 (73.9)	8 (100.0)
Expanding	25 (35.7)	6 (26.1)	0 (0.0)
Tumor extension			
Intrathyroidal	52 (74.3)	11 (47.8)	0 (0.0)
Extrathyroidal	18 (25.7)	12 (52.2)	8 (100.0)
LN metastasis			
No	47 (67.1)	22 (95.7)	4 (50.0)
Yes	23 (32.9)	1 (4.3)	4 (50.0)
Distant metastasis			
No	67 (95.7)	16 (69.6)	8 (100.0)
Yes	3 (4.3)	7 (30.4)	0 (0.0)

Supplementary Table S5: Expression of glutamine metabolism-related proteins according to p53 status in PDC and AC

Parameters	Total N=31 (%)	PDC and AC		
		p53 negative n=24 (%)	p53 positive* n=7 (%)	p-value
GLS1 (T)				0.054
Negative	9 (29.0)	9 (37.5)	0 (0.0)	
Positive	22 (71.0)	15 (62.5)	7 (100.0)	
GLS1 (S)				0.055
Negative	28 (90.3)	23 (95.8)	5 (71.4)	
Positive	3 (9.7)	1 (4.2)	2 (28.6)	
GDH (T)				0.325
Negative	3 (9.7)	3 (12.5)	0 (0.0)	
Positive	28 (90.3)	21 (87.5)	7 (100.0)	
GDH (S)				0.029
Negative	26 (83.9)	22 (91.7)	4 (57.1)	
Positive	5 (16.1)	2 (8.3)	3 (42.9)	
ASCT2 (T)				0.551
Negative	24 (77.4)	18 (75.0)	6 (85.7)	
Positive	7 (22.6)	6 (25.0)	1 (14.3)	

* p53 positivity defined as expression of more than 5% in tumor cells. PDC; poorly differentiated carcinoma, AC; anaplastic carcinoma
