# Supplementary material for: Expression of glutamine metabolism-related proteins in thyroid cancer
Source: Oncotarget. 2016 Jul 18;7(33):53628–41. doi: 10.18632/oncotarget.10682 (PMC5288210; doi:10.18632/oncotarget.10682)
Supplement: Supplementary file 2 [file oncotarget-07-53628-s002.doc]

| Supplementary table 2. Basal characteristics of patients with papillary thyroid carcinoma | | | | | | | |
| --- | --- | --- | --- | --- | --- | --- | --- |
| Parameters | Total  N=344 (%) | Histologic subtype | | p-value | BRAF V600E mutation status | | p-value |
|  |  | Conventional type  n= 304 (%) | Follicular variant  n= 40 (%) |  | No mutation  n= 106 (%) | Mutation  n= 238 (%) |  |
| Age (years) |  |  |  | 0.741 |  |  | 0.089 |
| <45 | 155 (45.1) | 136 (44.7) | 19 (47.5) |  | 55 (51.9) | 100 (42.0) |  |
| ≥45 | 189 (54.9) | 168 (55.3) | 21 (52.5) |  | 51 (48.1) | 138 (58.0) |  |
| Sex |  |  |  | 0.969 |  |  | 0.235 |
| Male | 68 (19.8) | 60 (19.7) | 8 (20.0) |  | 25 (23.6) | 43 (18.1) |  |
| Female | 276 (80.2) | 244 (80.3) | 32 (80.0) |  | 81 (76.4) | 195 (81.9) |  |
| Tumor size (cm) |  |  |  | 0.951 |  |  | 0.506 |
| ≤2.0 | 272 (79.1) | 240 (78.9) | 32 (80.0) |  | 80 (75.5) | 192 (80.7) |  |
| >2.0, ≤4.0 | 65 (18.9) | 58 (19.1) | 7 (17.5) |  | 23 (21.7) | 42 (17.6) |  |
| >4.0 | 7 (2.0) | 6 (2.0) | 1 (2.5) |  | 3 (2.8) | 4 (1.7) |  |
| Tumor margin |  |  |  | **0.002** |  |  | **0.004** |
| Infiltrative | 289 (84.0) | 262 (67.5) | 27 (67.5) |  | 80 (75.5) | 209 (87.8) |  |
| Expanding | 55 (16.0) | 42 (13.8) | 13 (32.5) |  | 26 (24.5) | 29 (12.2) |  |
| Tumor extension |  |  |  | 0.330 |  |  | 0.177 |
| Intrathyroidal | 106 (30.8) | 91 (29.9) | 15 (37.5) |  | 38 (35.8) | 68 (28.6) |  |
| Extrathyroidal | 238 (69.2) | 213 (70.1) | 25 (62.5) |  | 68 (64.2) | 170 (71.4) |  |
| Histologic subtype |  |  |  |  |  |  | **<0.001** |
| Conventional |  |  |  |  | 81 (76.4) | 223 (93.7) |  |
| Follicular |  |  |  |  | 25 (23.6) | 15 (6.3) |  |
| LN metastasis |  |  |  | 0.175 |  |  | 0.075 |
| No | 138 (40.1) | 118 (38.8) | 20 (50.0) |  | 50 (47.2) | 88 (37.0) |  |
| Yes | 206 (59.9) | 186 (61.2) | 20 (50.0) |  | 56 (52.8) | 150 (63.0) |  |
| Distant metastasis |  |  |  | 0.944 |  |  | 0.446 |
| No | 326 (94.8) | 288 (94.7) | 38 (95.0) |  | 99 (93.4) | 227 (95.4) |  |
| Yes | 18 (5.2) | 16 (5.3) | 2 (5.0) |  | 7 (6.6) | 11 (4.6) |  |
